# The PEX study – Exercise therapy for patellofemoral pain syndrome: design of a randomized clinical trial in general practice and sports medicine [ISRCTN83938749]

**DOI:** 10.1186/1471-2474-7-31

**Published:** 2006-03-17

**Authors:** Robbart van Linschoten, Marienke van Middelkoop, Marjolein Y Berger, Edith M Heintjes, Mark A Koopmanschap, Jan AN Verhaar, Bart W Koes, Sita MA Bierma-Zeinstra

**Affiliations:** 1 Department of General Practice, Erasmus MC, University Medical Centre, Rotterdam, The Netherlands; 2 Department of Orthopedics, Erasmus MC, University Medical Centre Rotterdam, The Netherlands; 3 iMTA, Erasmus MC, University Medical Centre Rotterdam, The Netherlands

## Abstract

**Background:**

Patellofemoral complaints are frequently seen in younger and active patients. Clinical strategy is usually based on decreasing provoking activities as sports and demanding knee activities during work and leisure and reassuring the patient on the presumed good outcome.

Exercise therapy is also often prescribed although evidence on effectiveness is lacking.

The objective of this article is to present the design of a randomized clinical trial that examines the outcome of exercise therapy supervised by a physical therapist versus a clinically accepted "wait and see" approach (information and advice about the complaints only).

The research will address to both effectiveness and cost effectiveness of supervised exercise therapy in patients with patellofemoral pain syndrome (PFPS).

**Methods/design:**

136 patients (adolescents and young adults) with patellofemoral pain syndrome are recruited in general practices and sport medicine centers. They will be randomly allocated receiving either 3 months of exercise therapy (or usual care.

The primary outcome measures are pain, knee function and perception of recovery after 3 months and 12 months of follow up and will be measured by self reporting.

Measurements will take place at baseline, 6 weeks, and 3 monthly until 1 year after inclusion in the study.

Secondary outcome measurements include an economic evaluation.

A cost-utility analysis will be performed that expresses health improvements in Quality Adjusted Life Years (QALYs) and incorporates direct medical costs and productivity costs

**Discussion:**

This study has been designed after reviewing the literature on exercise therapy for patellofemoral pain syndrome. It was concluded that to merit the effect of exercise therapy a trial based on correct methodological concept needed to be executed.

The PEX study is a randomized clinical trial where exercise therapy is compared to usual care. This trial started in April 2005 and will finish in June 2007. The first results will be available around December 2007.

## Background

Patellofemoral pain syndrome (PFPS) is a common complaint in adolescents and younger adults. Though exact epidemiological data do not exist, 5–6 six new cases per year in Dutch GP-practices may be expected [1]. The symptom most frequently reported is a diffuse peripatellar and retropatellar localized pain, typically provoked by ascending or descending stairs, squatting, cycling and sitting with flexed knees for prolonged periods of time [2,3]. Weakness of the knee extensors and abnormal firing patterns of the nerves innervating these knee extensors have been found in patients with PFPS. These phenomena are thought to cause maltracking of the patella through the femoral groove, resulting in increased intrapatellar pressure. Tight anatomical structures and heavy physical loading may add to the pressure. This pressure probably causes patellofemoral pain.

PFPS frequently becomes a chronic problem, forcing the patient to stop sports and other similar activities [4]. The long-term prognosis is generally more favorable for young patients, but seems to be independent of the presence of cartilage damage or gender [5].

Clinical guidelines of the Dutch College of General Practitioners [6] advise GPs to inform the patient about the background of the condition and its favorable prognosis. Patients are advised to refrain from all (sports-) activities that provoke pain, and to find alternative exercises to keep in shape. Non-weight bearing quadriceps strengthening exercises may be considered, but the guidelines explicitly mention that evidence for its effectiveness is lacking. Patellar taping is not advised. In case of prolonged unresponsive, severe complaints, referral to an orthopedic surgeon may be considered. General practitioners do not always adhere to these guidelines and prescriptions for analgesics such as paracetamol and NSAIDs to reduce pain and referrals to physical therapists (exercise therapy) are among the treatments regularly encountered (unpublished data authors). People involved in sports and athletic activities may consult sports clinics with their symptoms. In sports clinics it is more common to refer to physical therapy for exercises (unpublished data authors).

Evidence for the effectiveness of conservative therapies for PFPS is scarcely available [2]. Exercise therapy is based on the theoretical assumption that muscle weakness or imbalance is a major contributor in the development of PFPS. The recent Cochrane review [7] performed by our group identified only 3 trials comparing exercise therapy with a control group not receiving exercise therapy

We found limited evidence that exercise was beneficial, though the quality of the trials was such that further research was recommended to confirm this conclusion [7]. Recently a small placebo controlled trial was published investigating the short-term effectiveness of exercise therapy combined with taping and passive manual mobilization of the patella. The control group received sham ultrasound and placebo-taping. The authors reported beneficial effects in the intervention group (n = 33) compared the control group (n = 34) after 6 weeks follow-up [8]. Cost effectiveness data are not available at all.

Because physicians, especially the GP and sports physician, frequently are confronted with patients with PFPS, but by lack of evidence are unable to apply the most (cost)effective treatment, a randomized intervention study is highly indicated. The 'wait and see' policy advocated in the guidelines should be compared to the more active approach of exercise therapy under supervision of a physical therapist, in order to assess (cost)effectiveness of both approaches.

The trial will target patients (adolescents and young adults) presenting in general practice and sports clinics with the symptoms of PFPS and no history of previous active treatment with exercises.

In this article we will present the detailed protocol of the trial.

This trial started April 2005 and patients will be included until June 2006.

## Methods/design

### Study design

This study is a randomized clinical trial to study short-term and long-term (cost) effectiveness of exercise therapy in combination with advice and information on the background of PFPS compared to advice and information on the background of PFPS only ("wait and see")

**Figure 1:**
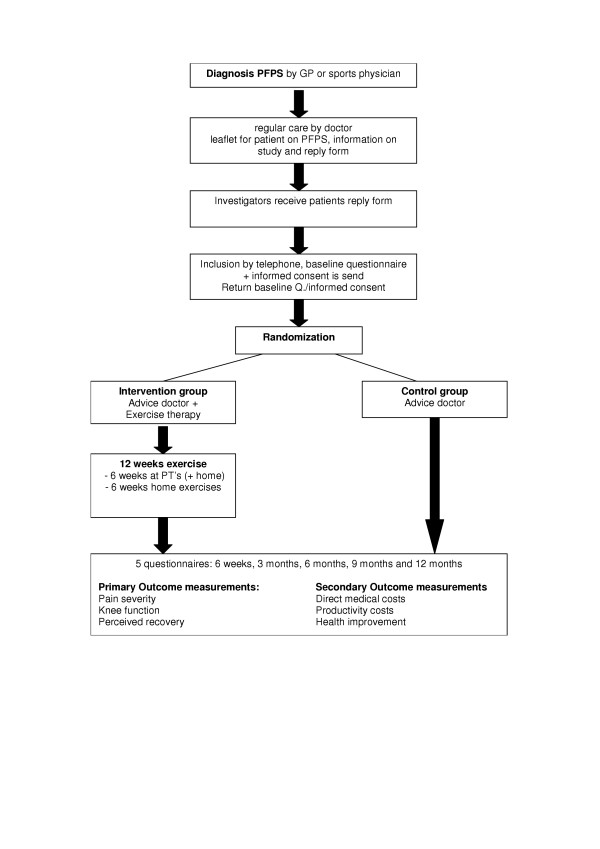
Flowchart of the study design.

The study design (Figure 1) was approved by the Medical Ethics Committee at the Erasmus MC – university medical centre Rotterdam. All patients gave written informed consent.

### Patient selection

Patients eligible for this trial are adolescents and young adults in the age of 14 to 40 years consulting the GP or sports physician for PFPS lasting longer than two months but not longer than 2 years. Recruitment will take place in "HONEUR" practices (a research network of 38 general practices allied with the Department of General Practice of Erasmus MC) and in 4 sports medical centers in Rotterdam, Leidschendam, Breda and Gorinchem.

The recruitment period is planned from April 2005 until June 2006.

### In- and exclusion criteria

Inclusion criteria are the following diagnostic criteria;

Presence of at least 3 symptoms of the following: pain when walking stairs, pain when squatting, pain when running, pain when cycling, pain when sitting with knees flexed for a prolonged period of time, grinding of the patella, positive physical tests (Clarke's test, Rabot sign, patella release test).

The exclusion criteria are: knee osteoarthrosis/arthritis, previous knee injury or knee operations, patellar tendinopathy, M. Osgood Schlatter, or other defined pathological conditions of the knee.

### Sample size

Sample size is based on studies included in our systematic review [7]. In a single study investigating a similar contrast of interventions [9] there was an absolute increase in recovery of 22% (19% recovery in the usual care group to 41% recovery in the exercise therapy group) after one year, OR 2.21 (95%CI 0.87 – 5.64)). This represents a clinically relevant increase and is expected to be even more pronounced after 3 months follow-up. Such a difference can be detected statistically (power 0.80, alpha 0.05 one-sided test) with 61 patients per group. With a potential dropout rate of 10% a total of 136 patients should be included.

### Intervention

The interventions that will be compared in this trial are:

A) Exercise therapy for a period of 6 weeks, provided by a physical therapist according to a standardized protocol drawn up according to present international expert opinion and modified by local participating physical therapists into a practical protocol which is feasible in daily practice. The program consists of static and dynamic muscular exercises for quadriceps muscles, balance exercises and flexibility exercises. Patients are directed to practice 7 times a week during 20 minutes.

**Figure 2:**
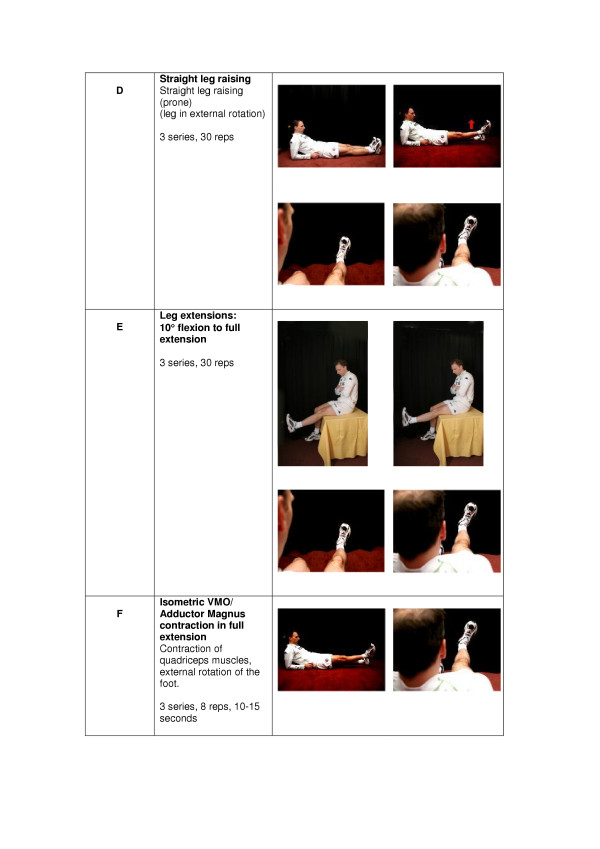
Sheet from the workout book with example exercises.

Instructions concerning the exercises will be noted on the "workout book" (Figure 2) which has been designed for the study. The notes regarding frequency and duration of exercises is send to the investigators after the three month period of exercise.

Patients will receive standardized information about the background and prognosis of PFPS on a specially designed leaflet. After the period of 6 weeks patients will be advised to keep up the exercises at home for the following 6 weeks.

B) The control group will receive the standardized information and advice. This advice consists of the information usually given by GPs, according to the guidelines: information about the background of the condition and its good prognosis, advice to refrain from all (sports-) activities that provoke pain, and to find alternatives to keep in shape.

An information leaflet for the patients has been compiled to contribute to the standardization for both groups.

#### Co-interventions

During the one year follow-up other interventions like the use of ice applications, bandages or braces, or consumption of oral analgesics (NSAIDs or paracetamol) indicated by pain severity are allowed for both groups. Information about these co-interventions will be collected after 6 weeks and every 3 months and during one year follow-up and will be used in the cost-effectiveness analysis.

### Randomization

After recruitment through the participating GP's and sport physicians the patient is finally accepted in the study after written informed consent and reassessment of the inclusion and exclusion criteria.

Following this informed consent and baseline assessments, patients are allocated to the intervention or control group using a blinded and computer based randomization list.

The randomization table will be stratified for the setting (general practice versus sports clinic) and for age (14–18 versus 19–40). Patient are informed about the treatment allocation and subsequently the patients in the exercise group receive their treatment from a physical therapist in a predetermined centre, whereas patients in the control group will not receive this intervention.

### Measurements

#### Outcome parameters

Primary outcome measures are: perceived recovery (measured with a 7 point Likert scale [10] functional disability using a disease-specific disability scale (Kujala Patellofemoral Scale) [11] and a pain severity using a numerical rating scale[12] after 3 and 12 months of follow-up. (Table 1)

Secondary outcome measures are cost-effectiveness after one year, the primary outcome parameters at 6 weeks follow-up, quality of life (Euroqol)[13] and a numerical rating scale (NRS 0–10) for difficulties encountered during work, school or sports activities. Medical consumption (visits to health care providers and consumptions of prescription or over the counter medication), absence from work or decreased productivity at work, and other indirect an direct costs are all included in the economic evaluation.

Baseline and follow-up questionnaires at 6 weeks and 3 and 12 months will be filled out by the patients themselves. Quality of life, direct costs and productivity costs and compliance to the interventions will be measured after 6 weeks and every 3 months during one year also by self report.

### Analyses

All analyses will take place after the trial has finished, no intermediate analyses will be performed.

To evaluate the effectiveness of supervised exercise therapy in patients with PFPS differences in clinical outcome measures between intervention and control group will be analyzed on the basis of intention to treat. Additionally, analysis per protocol will be conducted. Dichotomous outcomes at three and 12 months follow-up will be analyzed using logistic regression techniques and continuous outcomes with linear regression techniques. Of the primary outcomes perceived recovery will be dichotomized to recovered (fully, strongly) or not recovered (slightly-strongly worsened). Other primary outcome measurements will be analyzed as continuous variables

Analyses will be adjusted for baseline values and for co-interventions and possible prognostic factors in case the effect estimate changes with more than 10% when including these variables in the model.

Additionally the overall one year dichotomous outcomes will be analyzed using GEE (generalized estimating equations) [14], continuous outcomes will be analyzed using linear regression for repeated measurements. Both techniques take the correlation of multiple measurements within one patient into account.

Research question 2 "What is the cost-effectiveness of supervised exercise therapy in patients with PFPS...." is the basis for the economic evaluation.

In the economic evaluation (a cost utility analysis) both the costs and the consequences of both treatment options are compared and incremental costs and incremental health effects the latter (in terms of quality of life) is estimated.

The economic evaluation is performed alongside the randomized clinical trial. Patients will complete questionnaires for costs and quality of life after 6 weeks and every 3 months. Only if the difference in health between the treatment arms appears not to be stable over time, an additional modeling study using a Markov model will be performed. Statistical methods are used to describe uncertainty in costs and effects estimates based on patient data. A 95% confidence interval for the cost-utility ratio will be calculated and an acceptability curve will be presented

### Cost-analysis

For the economic evaluation a societal perspective is employed. The relevant costs are divided into direct medical costs and productivity costs.

#### - Direct costs

The costs of health care utilization during the twelve months follow-up consist of visits to a general practitioner, medical specialist, physiotherapist, manual therapist, prescribed and over the counter (OTC) medicines, alternative practitioners and hospitalization and appliances. In the patient questionnaire we ask for the health care consumption in the past six weeks. The costs for the period between two measurements (mostly 3 months) are established through linear interpolation. The medical consumption is valued based on resource costs and guideline costs [15]

#### - Productivity costs

The productivity costs are defined as the costs of absence from work due to PFPS, including the impact of compensation mechanisms [16] the costs of efficiency loss due to PFPS and the costs of hindrance at unpaid work. In the questionnaire the patients are asked to report the reason for absence from work and the number of absent days [17]. To this end we will use the PRODISQ questionnaire [18]. The valuation of an hour work the average productivity costs per hour worked will be based on the Net National Income per working hour[15]. The friction cost method is used to value the productivity costs related to paid work [19]. Productivity costs can also occur when people with health complaints are still working, but at a lower productivity level. This is called efficiency loss. The efficiency losses without absence are established by means of the Quality and Quantity-method [13]. Productivity losses at unpaid work are assessed by hindrance at unpaid work and the number of hours that housekeeping tasks were taken over by other people, and for how many hours paid help was needed. The costs of one hour of housekeeping tasks is set at the current price of one hour of simple professional home care.

#### - Patient outcome analysis in the economic evaluation

The patellar pain may affect Health Related Quality of Life. This is measured with a generic instrument, the EuroQol instrument EQ-5D [20,21] The EQ-5D descriptive system consists of five dimensions (Mobility, Self Care, Usual Activities, Pain/Discomfort and Anxiety/Depression) with three levels each (no problems, some problems and extreme problems), thus defining 243 (35) distinct health states. Respondents of the EuroQol EQ-5D describe their own health using this descriptive system. Preference weights based on the Time Trade-Off method for the 243 EQ-5D health states are available from a large-scale study in the UK[20,21]and a recent Dutch study [22] to calculate EQ-5D index scores that can be used as utilities to calculate QALYs.

## Discussion

The PEX-study has been designed after reviewing the literature on exercise therapy for patellofemoral pain syndrome [7]. It was concluded that though exercise therapy may have a beneficial effect on PFPS the scientific evidence is limited due to small sample size and a small amount of studies including a control group.

Based on available literature our research group expects to discover (beneficial) changes from exercise therapy in perceived recovery, pain severity and functional disability in the PEX study.

The PEX study is a randomized clinical trial where exercise therapy is compared to usual care. The trial started in April 2005 and is expected to finish in June 2007. The first results will be available around December 2007.

## Competing interests

The author(s) declare that they have no competing interests.

## Authors' contributions

SMABZ, BWK and EMH conceived of the study, developed the design of this trial and contributed to the content of the article. MAK, JANV and MYB participated in the design of the study. RL participated in the design and coordination of the study and wrote the article. MM coordinates the trial and is responsible for data acquisition.

All authors read and approved the final article.

## Pre-publication history

The pre-publication history for this paper can be accessed here:



## Figures and Tables

**Table 1:** Questionnaires for primary and secondary outcome measurements

**Primary outcome measurements**		
Perceived recovery	-	Likert scale
Functional disability	-	Kujala Patellofemoral Scale
Pain Severity	-	Numerical Rating Scale (NRS)
**Secondary outcome measurements**		
Direct medical costs	-	Healthcare consumption
Productivity costs	-	PRODISQ
Health(improvement)	-	EuroQol
